# HIV and the tuberculosis “set point”: how HIV impairs alveolar macrophage responses to tuberculosis and sets the stage for progressive disease

**DOI:** 10.1186/s12977-020-00540-2

**Published:** 2020-09-23

**Authors:** Sara C. Auld, Bashar S. Staitieh

**Affiliations:** 1 Emory University School of Medicine, Atlanta, GA USA; 2 Rollins School of Public Health, Emory University, Atlanta, GA USA

**Keywords:** Tuberculosis, HIV, Macrophages, Coinfection, Innate immunity

## Abstract

As HIV has fueled a global resurgence of tuberculosis over the last several decades, there is a growing awareness that HIV-mediated impairments in both innate and adaptive immunity contribute to the heightened risk of tuberculosis in people with HIV. Since early immune responses to *Mycobacterium tuberculosis* (*Mtb*) set the stage for subsequent control or progression to active tuberculosis disease, early host–pathogen interactions following *Mtb* infection can be thought of as establishing a mycobacterial “set point,” which we define as the mycobacterial burden at the point of adaptive immune activation. This early immune response is impaired in the context of HIV coinfection, allowing for a higher mycobacterial set point and greater likelihood of progression to active disease with greater bacterial burden. Alveolar macrophages, as the first cells to encounter *Mtb* in the lungs, play a critical role in containing *Mtb* growth and establishing the mycobacterial set point. However, a number of key macrophage functions, ranging from pathogen recognition and uptake to phagocytosis and microbial killing, are blunted in HIV coinfection. To date, research evaluating the effects of HIV on the alveolar macrophage response to *Mtb* has been relatively limited, particularly with regard to the critical early events that help to dictate the mycobacterial set point. A greater understanding of alveolar macrophage functions impacted by HIV coinfection will improve our understanding of protective immunity to *Mtb* and may reveal novel pathways amenable to intervention to improve both early immune control of *Mtb* and clinical outcomes for the millions of people worldwide infected with HIV.

## Introduction

Although tuberculosis incidence declined worldwide over the course of the 20^th^ century [1–4], these gains were quickly reversed with the onset of the HIV epidemic in the 1980s [5]. Tuberculosis is now the leading global killer among infectious diseases and the leading cause of death among people with HIV [6]. The profound T cell depletion induced by HIV has long been recognized as a significant driver of the tuberculosis epidemic in people with HIV [7, 8]. However, there is growing awareness that HIV-mediated impairments in innate immunity also contribute to the increased risk of tuberculosis in this vulnerable population. These HIV-mediated impairments in innate immunity are particularly relevant early in *Mycobacterium tuberculosis (Mtb)* infection, prior to the onset of adaptive immune responses [9] and, by impairing early control of *Mtb*, set the stage for accelerated progression to active tuberculosis disease.

**Fig. 1:**
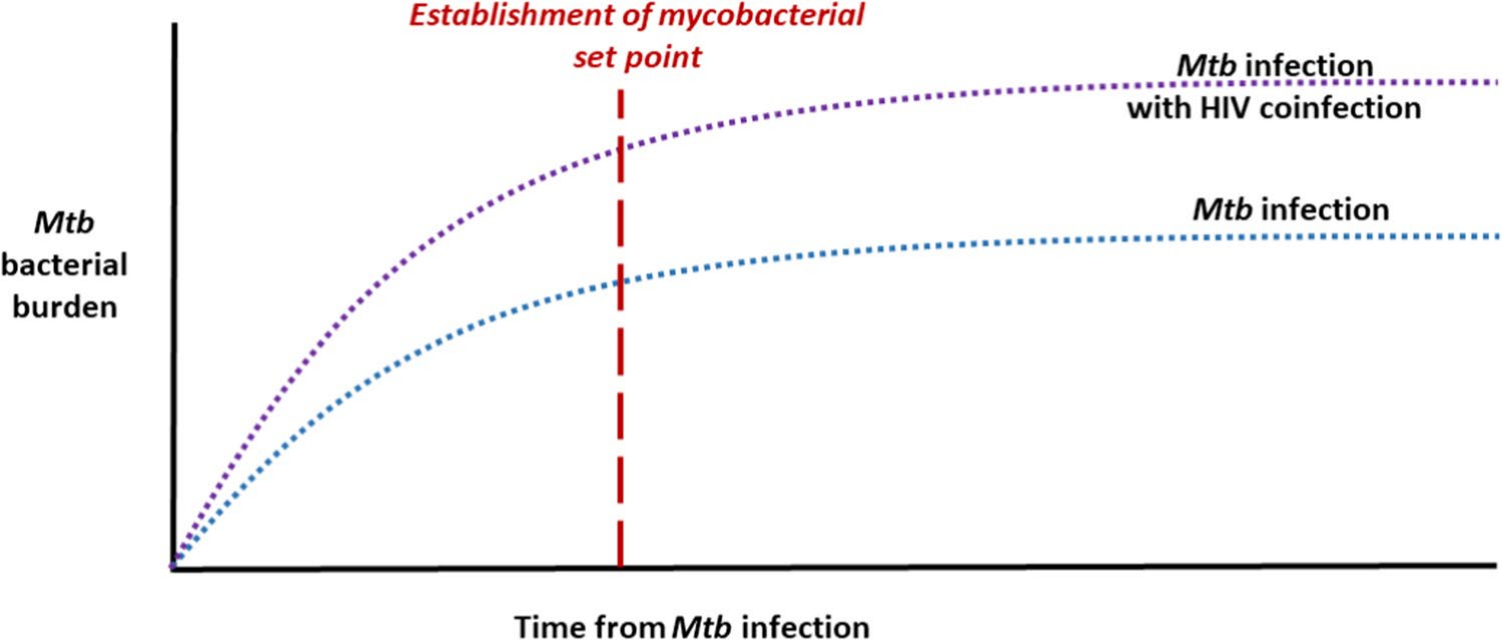
Graphical depiction of the mycobacterial set point as a theoretical construct, represented as the *Mycobacterium tuberculosis* (*Mtb*) bacterial burden after infection, either alone or in the context of HIV coinfection. After infection, *Mtb* bacterial burden increases until adaptive immunity is activated, thereby establishing the mycobacterial set point. Alveolar macrophages, the first cells to encounter *Mtb*, are an important component of the early innate immune response to *Mtb*. As reviewed in the main text, the mycobacterial burden at the point of adaptive immune activation plays a important role in the subsequent control of infection and progression to active tuberculosis disease. In the case of HIV coinfection, impairments in alveolar macrophage functions lead to poor early immune control of *Mtb* which, in turn, leads to a higher mycobacterial set point and greater *Mtb* bacterial burden at the time when adaptive immunity is activated

The pattern of early immune control setting the stage for longer term control—and clinically meaningful endpoints—parallels our understanding of the HIV set point. It was first recognized in the late 1990s that the HIV viral load soon after seroconversion, i.e., the HIV set point, is predictive of disease progression [10, 11]. Following infection, HIV replicates unchecked until an initial, albeit ineffective, host immune response develops and the set point is established [12–14]. When viewed through this same lens, early host–pathogen interactions following *Mtb* infection can be thought of as establishing a “mycobacterial set point” (Fig. 1). Unlike the set point in HIV, which occurs after an ineffective adaptive immune response, the mycobacterial set point is established before the onset of adaptive immunity.

Although we currently lack the tools to measure the mycobaterial set point clinically, this theoretical construct provides a framework for understanding early immune responses to *Mtb*, particularly those of the alveolar macrophage. While a multitude of cell types, including recruited interstitial macrophages, dendritic cells, and neutrophils, are infected prior to the onset of adaptive immunity [15], work by Cohen and others has confirmed the importance of the alveolar macrophage to the early *Mtb* response [16]. As we will emphasize in this review, a focus on this critical early period and the mycobacterial set point are useful in understanding the relative impacts, both on a cellular and clinical level, of various host and pathogen impairments that occur in the context of coinfection with *Mtb* and HIV. The concept of a mycobacterial set point also provides us with an overall index of immune health in the context of HIV/*Mtb* coinfection and thereby offers a useful gauge of efficacy when specific pathways are manipulated for therapeutic benefit. Host immunity to *Mtb* remains poorly understood, but a focus on the role of alveolar macrophages in establishing a mycobacterial set point and on alterations in macrophage control in the context of HIV coinfection has the potential to yield great insight into some of the earliest immune events in *Mtb* infection.

Although *Mtb*- and HIV-mediated impairments in pulmonary innate immunity and alveolar macrophage function have been reviewed elsewhere [17, 18], the impact of these impairments in the context of coinfection has not been well described. In this review, we will explore how HIV coinfection makes alveolar macrophages, the first cells to encounter *Mtb*, more permissive of early mycobacterial growth and is therefore likely to increase the mycobacterial burden at the point at which adapative immunity is activated and the mycobacterial set point is established [16]. By curtailing early immune control and delaying the onset of adaptive immunity, HIV coinfection leads to a higher mycobacterial set point and, consequently, a greater bacterial burden (Fig. 1).

In order to demonstrate the utility of the mycobacterial set point framework, we will first review epidemiologic data supporting the importance of innate immunity in people with tuberculosis and HIV coinfection. We will then discuss some of the model systems that are used to study macrophage responses to *Mtb* and HIV and animal model findings in support of a mycobacterial set point. We will then highlight existing data on alveolar macrophage functions—from macrophage phenotype, key cytokines, and oxidative stress, to pathogen recognition and phagocytosis, cell death pathways, and activation of adaptive immunity—that are impacted by either *Mtb* infection, HIV infection, or coinfection. The examples we provide, which come from a range of cell types and organ systems, including primary alveolar macrophages, monocyte-derived macrophages, and cell lines, are not meant to be exhaustive or comprehensive. Rather, our goal is to provoke further inquiry and investigation into this critical area by offering a framework by which to contextualize studies in the field. We will close the review by highlighting several promising avenues for studying the mycobacterial set point in humans, in order to underscore the potential benefit of this concept as a framework for clinically relevant research.

### Epidemiologic data for the role of innate immunity in HIV and tuberculosis

From the earliest days of the HIV epidemic it has been clear that tuberculosis outcomes were markedly worse in people with HIV. In a series of nosocomial tuberculosis outbreaks among people with HIV in the early 1990s, the overwhelming majority of patients died, often within a month or two of their diagnosis [19–21]. These individuals had very advanced HIV, which undoubtedly contributed to their high mortality rates [21]. However, their rapid progression from exposure to active disease, in a time frame prior to the expected onset of adaptive immunity, suggests that impaired innate immunity was likely a contributing factor. Similarly, in a study of South African gold miners newly infected with HIV, the risk of tuberculosis disease was found to increase very early in the course of HIV infection, when CD4 counts would still be in a normal range [22]. Another study from South Africa found that people with HIV on antiretroviral therapy (ART) with normal CD4 counts had four times the risk of tuberculosis than people without HIV [23]. Likewise, in Italy, a setting with a low burden of both HIV and tuberculosis, nearly a third of patients diagnosed with HIV-associated tuberculosis in one study had been receiving ART for a median of 27 months, at which point CD4 counts would typically have reached a normal range [24]. Taken together, these data offer epidemiologic evidence that defects in both innate and adaptive immunity are likely to contribute to the greater risk of tuberculosis in people with HIV. While these clinical observations cannot establish mechanism or causality, they do provide a rationale for pursuing further basic science and translational studies to better understand the role of innate immunity in controlling *Mtb* infection.

### Model systems for studying alveolar macrophage responses to Mtb and HIV

Over the last century, scientists from successive eras have made remarkable discoveries by bringing the newest available techniques to bear on their investigations of *Mtb*, with various model systems employed over time. While each system has distinct advantages and disadvantages in addressing particular questions, the sheer volume of data derived from the many available systems can make it hard to develop a coherent narrative regarding *Mtb* infection in humans. Although a full review of these features of the tuberculosis literature is beyond the scope of this review, we will offer a brief overview of the main characteristics of some of the major models to provide context for the reader [25, 26].

Despite their inability to model sophisticated cellular interactions, in vitro systems have long been used as tools for studying *Mtb* [27]. A variety of myeloid cell lines have been employed, and are often helpful in quantifying cytokine and other signaling responses to *Mtb*. In addition, due to the ease with which in vitro systems can be manipulated, studies with primary macrophages from animal models have enabled investigators to dissect the roles of individual proteins and pathways in the *Mtb* response [28, 29]. Finally, human primary cells have been used in a variety of ways, including induced pluripotent stem cells, human macrophages, granuloma models, and many more. Each system has proven useful in addressing different aspects of the *Mtb* response, but all are hampered by their inability to coordinate the full spectrum of in vivo responses. An additional challenge is the conflation of different types of macrophages, whether from cell-lines, animal models, or humans, as a homogenous population. Rather, there is a growing appreciation of the distinctions between tissue-derived and bone-marrow derived macrophages, from their ontogeny to functional capacity and self-maintenance [30–32]. These distinctions are highly relevant to this review, as bone-marrow derived macrophages do not become polarized in the same manner as alveolar macrophages following *Mtb* exposure, and thus cannot be expected to behave the same [33]. However, given challenges in obtaining primary alveolar macrophages, particularly from humans [34], many studies are conducted with derived cells.

With regards to animal systems, ease of use in handling the zebrafish-*Mycobacterium marinum* model has made it a powerful tool for the study of *Mtb* [35]. Several key components of the human response to *Mtb* are present in zebrafish, including the leukotriene A4 hydrolase locus, and it has been used to study everything from treatment strategies for drug-resistant *Mtb* to vaccine development. The mouse model has the virtue of capturing vertebrate, mammalian immune responses in a reproducible, easily scalable system. However, different mouse strains have different levels of susceptibility to *Mtb* and mice do not typically form granulomas or cavities [36]. As a result, the mouse model tends to be suited for studies of cell-mediated immunity and drug development, but less useful for studies of human clinical outcomes [37]. Guinea pigs provide another small animal model, with the benefit of recapitulating lung necrosis and other human features, but challenges in measuring their immune responses has historically limited their widespread use [25]. Rabbits have also provided an effective model system for *Mtb* research, largely due to the fact that the structural similarities between their lungs and human lungs leads to similar pathology in response to *Mtb*. That said, their larger size and the greater resources required to utilize them has limited their broad applicability [36, 38]. Finally, non-human primates, particularly rhesus and cynomolgus macaques, have marked genetic and immunologic similarities to humans and, as such, have yielded significant advances in our understanding of host–pathogen interactions and the potential for vaccine-induced protection in recent years [39, 40]. However, given the vast resources needed to sustain these animals, there are a limited number of research centers capable of conducting non-human primate studies.

### Evidence to date for a “mycobacterial set point”

While in-depth analysis of early immunologic events in human *Mtb* infection remains challenging for the reasons discussed above, Poulsen and others established over 50 years ago that tuberculin skin test (TST) conversion in humans, an indicator of the onset of the adaptive immune response, generally occurred by 6 weeks after exposure [41–43]. Poulsen also determined that this period prior to TST conversion is not a time of immunologic quiescence; rather, many individuals had evidence of a robust inflammatory response with fever and elevated inflammatory markers.

Since those landmark studies, much of our understanding of these early events has relied on data from animal models. Experiments in rabbits and guinea pigs by Lurie, Smith, and Harding, established that macrophages provide a niche within which *Mtb* multiplies logarithmically during early infection [44, 45]. Others have demonstrated that the onset of adaptive immunity in response to *Mtb* is indeed delayed, arising in part from delayed activation of CD4+ T cells within local lymph nodes [46, 47]. This delay of several weeks, as compared to several days with organisms such as *Salmonella typhi* and *Listeria monocytogenes*, translates into relatively unchecked bacterial growth during the early phase of infection [48, 49]. After this period of logarithmic growth, which has been demonstrated in rabbits, mice, and guinea pigs, the bacteria enter a relatively stationary phase [50]. During this critical time, *Mtb* subverts alveolar macrophage defenses, thereby ensuring prolonged intracellular survival and mycobacterial growth within this pulmonary niche. Whether this failure of immunity represents an intrinsic deficiency of macrophages or an insufficient activation of macrophages by T cells remains uncertain [51].

As discussed above, there has been a proliferation of work with non-human primate models in recent years that shed further light on the establishment of the set point, particularly with regards to granuloma formation [52]. Studies of macaques with aerosol exposure to *Mtb* have found that early mycobacterial dissemination, as reflected by the development of new granulomas in the first 3–6 weeks following infection (i.e., the period during which the set point is established), is associated with subsequent progression to active disease [53, 54]. Conversely, the absence of new granuloma formation, which could be construed as a “low” set point, is associated with immune control and long-term maintenance of latent infection. Granuloma size can also stratify the risk of dissemination, with larger granulomas associated with a greater potential for future mycobacterial spread [55]. By linking early immunologic events to clinically meaningful outcomes, these findings underscore the critical importance of early immune control prior to the onset of adaptive immunity. They also provide evidence to support the longstanding belief that progression to tuberculosis disease is determined at the level of the individual granuloma.

### Alveolar macrophage phenotypes in Mtb infection

As the first cell type to encounter *Mtb*, the alveolar macrophage is a key player in the early stages of infection and *Mtb* is known to both cause and benefit from certain alterations in macrophage functionality [56]. Several excellent reviews have recently examined the heterogeneity and plasticity of macrophages, in addition to proposing a uniform approach to macrophage nomenclature [31, 32]. Alveolar macrophages play myriad roles in the lungs, from housekeeper to innate immune effector, and their role depends heavily on the microenvironment within the alveolar space. For example, in response to inflammatory stimuli such as lipopolysaccharide (LPS), alveolar macrophages develop an inflammatory phenotype with increased production of cytokines such as TNF-α and IL-6. Conversely, in the setting of IL-4 exposure, alveolar macrophages shift to an immunosuppressive phenotype and increase production of regulatory cytokines such as TGF-β [31]. Although debate continues as to how best to characterize macrophages and how dynamically tissue resident subtypes respond to their local cytokine milieu, it is well known that macrophages respond to different cytokine programs with a variety of functional changes. Most experts agree that these changes exist on a spectrum between the classically activated (or M1, or inflammatory) macrophage and an alternatively activated (or M2, or regulatory) macrophage.

**Fig. 2:**
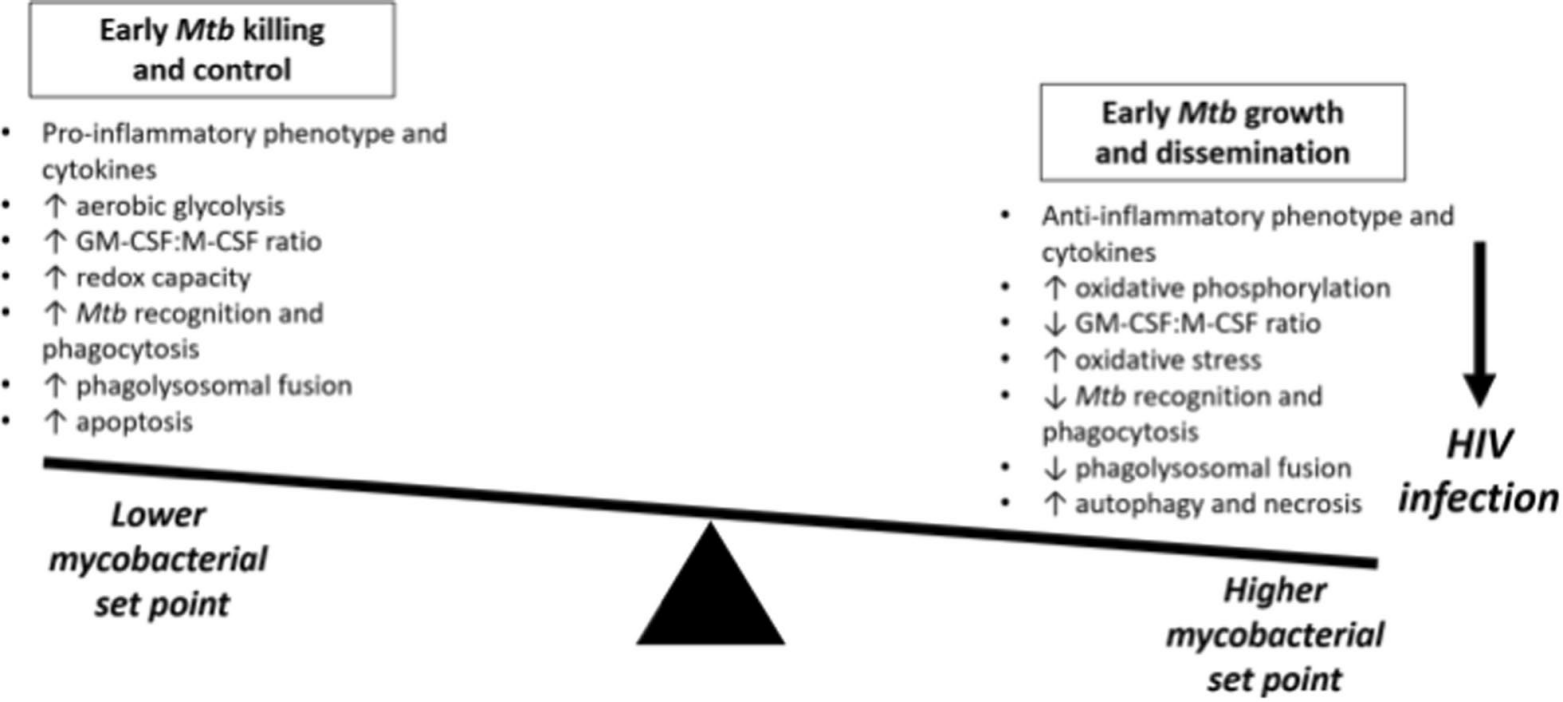
The balance of alveolar macrophage polarization and activity. Early *Mtb* killing and control leads to a lower mycobacterial set point, whereas early *Mtb* growth and dissemination lead to a higher mycobacterial set point. HIV infection favors early *Mtb* growth and dissemination and a higher mycobacterial set point

In the context of *Mtb* infection, the “macrophage polarization ratio,” an indicator of the balance between pro-inflammatory and anti-inflammatory genes, has been employed to explore how macrophage functionality and subsequent granuloma formation affects the probabilities of *Mtb* control and dissemination (Fig. 2) [57]. With this approach, NF-kB signaling was found to be an essential component of the classical activation profile, which, when triggered early, leads to improved clinical tuberculosis outcomes in a nonhuman primate model [57].

Alveolar macrophages are also severely impacted by the presence of HIV infection and have been shown to be a reservoir for the virus within the lungs [58]. Importantly, coinfection shifts macrophage polarization in a fashion favorable for pathogen survival [59]. Both HIV and *Mtb* stimulate a cytokine milieu in the alveolar space that pushes macrophages into an inflammatory phenotype optimized for the uptake of foreign microbes. Although these inflammatory cells would ordinarily excel at pathogen killing, the intracellular effects of HIV and *Mtb* render the macrophages less effective and, as a result, permit ongoing pathogen growth [60]. At the same time, there is ample evidence that *Mtb* infection of alveolar macrophages creates a permissive environment for HIV infection and replication, through a variety of mechanisms including increased surface expression of the HIV receptors CCR5 and CXCR4, increased HIV transcription and replication, and induction of nanotubes that promote cell-to-cell HIV viral spread between macrophages [61–64]. Taken together, this synergy between *Mtb*- and HIV-mediated impacts on macrophages enables intracellular growth and survival of both pathogens and, more broadly, fuels the global syndemic of coinfection.

Macrophage function and polarization is also influenced by immunometabolism, the balance of cellular energy utilization. This represents another growing area of inquiry in which *Mtb* and HIV are likely to have complementary, yet deleterious, effects. *Mtb* infection of alveolar macrophages has been shown to shift cellular metabolism from oxidative phosphorylation to aerobic glycolysis, a shift associated with a more inflammatory, M1-like phenotype [65]. This shift triggers an increase in cellular IL-1β production along with a decrease in IL-10 production, which, in concert, should increase bacillary killing in vitro. Yet, other investigations suggest that persistence of fatty acid oxidation in alveolar macrophages may permit ongoing intracellular *Mtb* growth [66]. At the same time, HIV infection also alters cellular energy balance, oxidative stress levels, and mitochondrial bioenergetics, including for infected macrophages [67–70]. Further study of the combined, net effects of *Mtb* and HIV on alveolar macrophage immunometabolism is needed to expand our understanding of how coinfection impairs early mycobacterial control.

### Cytokine expression and innate immunity in Mtb and HIV

Macrophage cytokine expression profiles may also vary according to the stage of *Mtb* infection, whereby the secreted protein ESAT-6 initially skews macrophages towards a M1 phenotype, potentially to facilitate the establishment of a granuloma, and then later to a more permissive M2 phenotype [71]. IFN-γ, a paradigmatic pro-inflammatory cytokine, drives macrophages towards an inflammatory phenotype and, under normal circumstances, is critical for *Mtb* control. However, peripheral blood mononuclear cells isolated from people with HIV have depressed IFN-γ production in response to purified protein derivative (PPD) [72, 73]. IL-4, another key cytokine for macrophage polarization, is induced by HIV glycoprotein (gp) 120 [74]. This induction shifts macrophages to an anti-inflammatory phenotype, potentially further reducing early *Mtb* control. In people without HIV, elevated IL-4 levels have been associated with progression to tuberculosis disease and the development of pulmonary cavities, thus underscoring the clinical impact of these shifts [75–77]. Interestingly, the relative balance of these cytokines may vary according to the stage of HIV infection, which could translate into differential susceptibility to and control of *Mtb* infection [60].

Several key cytokines important for both macrophage polarization and the response to *Mtb* are also altered in the presence of HIV coinfection. For example, GM-CSF plays an essential role in cellular control of *Mtb* [78, 79] and its activity is impaired in monocyte-derived macrophages infected with HIV [80]. The GM-CSF:M-CSF balance is also altered by the presence of *Mtb* [81] and M-CSF-driven macrophages are more permissive to *Mtb* replication and dissemination [82]. Similarly, interleukin-1 receptor-associated kinase M (IRAK-M), which is downstream of the GM-CSF-dependent transcription factor PU.1, correlates directly with mycobacterial load in human lung tissue and impacts *Mtb* survival [83]. Thus, the combined impact of *Mtb* and HIV on the balance of cytokines within the lungs leads to an environment that permits the growth of both pathogens.

### Alveolar macrophage oxidative stress in Mtb and HIV

Another avenue ripe for further exploration is the effects of HIV and *Mtb* on redox systems, which can also modulate macrophage function [84–86]. HIV is known to impair antioxidant defenses in the alveolar macrophage and raise levels of oxidative stress in the lung more generally [18, 87]. The effects in the lung are likely due, at least in part, to glutathione depletion in the airways of people with HIV [88, 89]. *Mtb*, on the other hand, has successfully adapted to hostile intracellular conditions, including high oxidative stress [90, 91]. *Mtb* may also directly contribute to oxidative stress by inducing heme oxygenase (HO)-1, the levels of which directly correlate with treatment outcomes in people with active tuberculosis disease [92]. Several studies have demonstrated improved *Mtb* control with glutathione supplementation, including one study using macrophages isolated from people with HIV in which supplementation led to enhanced inflammatory cytokine production and restored macrophage capacity to control *Mtb* growth [89, 93–97]. Of note, the oxidative stress induced by Mtb-infected macrophages is also likely to promote HIV reactivation, further complicating the relationship between these two pathogens [98].

### Mtb recognition and phagocytosis by alveolar macrophages in HIV

**Fig. 3:**
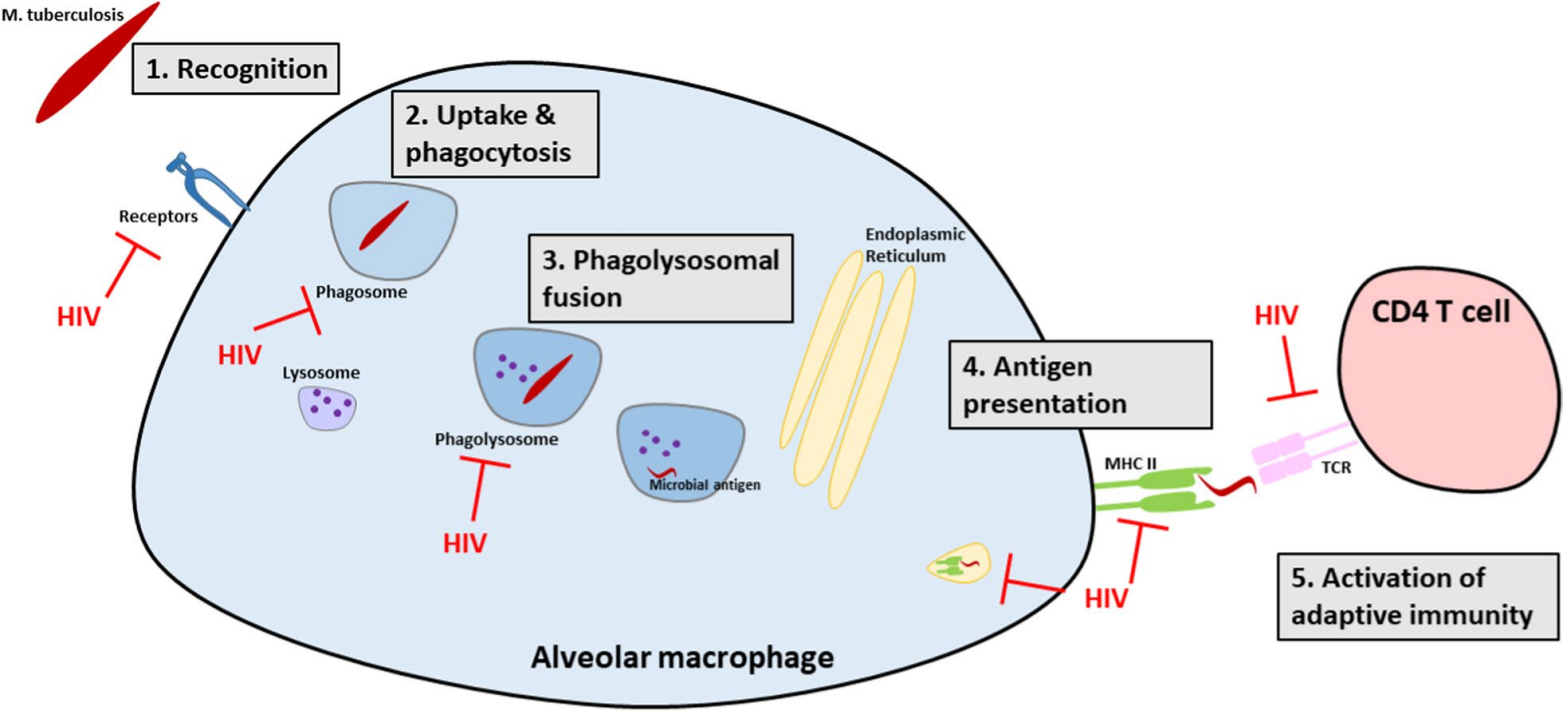
Events in alveolar macrophage uptake and response to *Mtb* infection that are impacted by HIV coinfection. (1) Recognition: HIV coinfection downregulates alveolar macrophage expression of *Mtb* recognition receptors including mannose and toll-like receptors. (2) Uptake and phagocytosis: HIV coinfection impairs phagocytosis. (3) Phagolysosomal fusion: HIV coinfection disrupts endosomal trafficking and impairs phagolysosomal maturation. (4) Antigen presentation: HIV coinfection leads to the expression of immature MHC class II complexes and thereby impairs the activation of adaptive immune responses. (5) Activation of adaptive immunity: HIV coinfection reduces the expression of costimulatory molecules for activation of adaptive immunity. The net effect of these HIV-mediated impairments is to enable increased intracellular and extracellular *Mtb* growth which ultimately leads to a higher mycobacterial set point

An important function of macrophages in the alveolar space is the differentiation of friend versus foe via pathogen recognition and phagocytosis (Fig. 3) [99–101]. Particularly germane to the recognition of *Mtb* is the mannose receptor, which is downregulated in people living with HIV [102]. HIV also leads to reduced expression and activity of toll-like receptors (TLR), another key receptor for macrophage pathogen recognition [103–105]. When combined with shifts in TLR-mediated regulation of nitric oxide production induced by *Mtb* [104], these impairments have the potential to reduce *Mtb* uptake by alveolar macrophages and facilitate prolonged extracellular growth.

Once a pathogen like *Mtb* has been recognized, it then undergoes phagocytosis [99, 100]. HIV creates favorable conditions for bacteria by impairing phagocytosis of pathogens including *Streptococcus* [106]. The HIV protein Nef has also been shown to interfere with phagosome formation by impeding endosomal recruitment and remodeling [107]. Of note, impaired phagocytosis has been observed even in alveolar macrophages not directly infected by HIV [58, 108]. While phagocytosis of *Mtb* in the setting of HIV has not specifically been studied, such impairments would enable further extracellular growth [18, 109].

HIV has also been found in macrophage endosomes and is known to impair phagolysosomal fusion in the macrophage [110, 111]. At least some of these impairments may be mediated through the effects of the HIV-related protein transactivator of transcription (Tat) [112]. Another HIV protein, Vpr, has also been shown to perturb phagolysosomal maturation by altering microtubule-dependent trafficking within macrophages [113]. In addition, HIV has been shown to co-opt the Rag GTPases, resulting in further disruption of endosomal trafficking [114]. This disruption may facilitate HIV replication and simultaneously assist *Mtb* evasion of lysosomal fusion and killing. IL-10 has also been shown to dysregulate the response to *Mtb* in the setting of HIV, at least in part by impairing phagosome maturation [115, 116]. However, in one study of alveolar macrophages from humans with active tuberculosis disease, phagolysosomal acidification was equally disrupted among those with and without HIV, highlighting existing uncertainties about the impacts of *Mtb* and HIV on alveolar macrophage phagocytosis [117]. Thus, although further research is needed to define the relevant mechanisms by which HIV impairs the phagocytic response to *Mtb*, the bulk of the existing data suggest that endosomes fail to fuse with acidified lysosomes, thereby promoting *Mtb* survival [118].

### Cell death pathways in Mtb-HIV infected macrophages

Alveolar macrophage cell death pathways range from apoptosis to autophagy and necrosis. *Mtb* is known to shift the balance toward necrosis by actively inhibiting apoptosis, thereby allowing the extracellular release of viable mycobacteria from dying alveolar macrophages [119, 120]. Similarly, HIV, via the viral proteins Tat and glycoprotein-120 (gp-120), stimulates the triggering receptor expressed on myeloid cells-1 (TREM-1), thereby inhibiting apoptosis in a p65-dependent manner [121]. HIV may also directly induce autophagy through the Nef protein, in order to modulate viral replication and survival, albeit in a manner that may be synergistic with *Mtb*-mediated inhibition of apoptosis [122, 123]. Gp-120 also impairs apoptosis-associated killing of pneumococcus within alveolar macrophages, indicating that this impairment may not be specific to *Mtb* [124]. Impaired apoptosis of *Mtb* has been tied by other investigators to IL-10, which is increased in the bronchoalveolar lavage fluid of people with HIV [125]. Additional research is needed to further delineate the impact of these shifts in cell death pathways and to identify whether there are therapeutic opportunities to improve *Mtb* control in patients with and without HIV. One promising recent report found that all-trans retinoic acid was able to promote autophagy of human alveolar macrophages in the setting of *Mtb* infection [126].

### Activation of adaptive immunity in Mtb-HIV coinfection

The final step required for establishment of the mycobacterial set point is activation of the adaptive arm of the immune system (Fig. 3). *Mtb* has been shown to delay the activation of adaptive immunity in mice by slowing bacterial transport to local lymph nodes [46]. This delay allows for a prolonged period of unchecked bacterial growth within the lungs, with a 10,000–100,000-fold expansion of the *Mtb* pulmonary population during this time. At least one driver of this delay is *Mtb*-mediated inhibition of MHC class II antigen presentation by dendritic cells [127]. *Mtb* also manipulates antigen presentation by diverting bacterial proteins through a vesicular export pathway, away from MHC class II presentation. In the context of HIV, the viral protein Nef has been shown to promote the expression of immature, functionally incompetent MHC class II complexes [128, 129]. Recent data have more directly linked these two by demonstrating that HIV-infected dendritic cells have reduced ability to upregulate key costimulatory molecules critical for antigen presentation (e.g., CD40, CD80, and CD86) in the presence of *Mtb* infection [130]. All of this would combine to delay the onset of adapative immunity and further increase the mycobacterial set point.

### Net effects: the combined immunologic consequences of coinfection with Mtb and HIV

As should be clear from the preceding sections, studies detailing the effects of *Mtb* and HIV on the immune responses within the alveolar space have found an abundance of affected cytokines, pathways, and response elements. Although much of the available data suggests ways in which *Mtb* and HIV each make it more likely for the other to gain ground within an individual’s lungs, the myriad as-yet-unstudied pathways leave open the distinct possibility that some of their particular effects may cumulatively cancel out those of the other pathogen. It is in this key domain that we believe the concept of a mycobacterial set point most clearly demonstrates its worth. Because the set point relates to the mycobacterial burden at the moment in which the adaptive immune system activates, it offers a clinically relevant marker that synthesizes the various effects of coinfections, comorbidities, and therapeutics. As a research tool, then, it will help us understand whether the net effect of a given intervention actually reduces the overall mycobacterial burden, as opposed to fruitfully manipulating a single pathway (with the knowledge that doing so may have deleterious effects on others).

### Measuring the mycobacterial set point in human infection and disease

Fortunately, several novel approaches provide hope for expanding our understanding of these early immunologic events in humans. These tools have the potential to vastly enhance our current knowledge by quantifying the functional impact of coinfection on bacterial burden and early human immune responses, while also offering insight into mechanisms underlying early immune control or escape.

The first such tool is 2-deoxy-2-[18F]fluoro-d-glucose ([18F]FDG) positron emission tomography combined with computed tomography (PET–CT), which identifies areas of increased cellular metabolic activity as would occur with an immune response to infection. Several macaque studies have leveraged PET-CT imaging to gain insight into early infection by following the progression of individual granulomas after aerosol infection [53, 54, 131, 132]. PET-CT has also been employed to study tuberculosis in humans, including a cohort of 35 people with HIV and latent tuberculosis infection, 10 of whom had PET-avid lesions suspicious for subclinical disease [133]. Those with PET activity were significantly more likely to develop active tuberculosis disease during the following 6 months, suggesting metabolic activity on PET imaging may be able to identify patients with ineffective early immune responses to *Mtb* infection.

Blood-based immunologic signatures represent another potential avenue for studying early infection. Recent studies in macaques and humans have identified RNA signatures predictive of progression to active tuberculosis disease [134–136]. Notably, the macaque signature was predictive just 3–6 weeks after aerosol infection, providing compelling evidence for the value of these signatures even very early in infection. This signature also identified a novel gene in tuberculosis pathogenesis, *PRDX2*, a member of the reactive oxygen species scavenger system. Further studies are warranted to explore the role of *PRDX2* in early infection, and how that response may be skewed by HIV and other known tuberculosis risk factors. Similarly, characterization of *Mtb*-specific T cell responses may provide another means for understanding adaptive immune responses important in early infection. In a recent report of a whole blood assay of T cell responses to 60 *Mtb* antigens, immunogenic *Mtb* antigens were successfully identified across a diverse range of populations and infectious states [137]. Longitudinal studies are underway to determine if these T cell signatures correlate with early immune responses—both those that clear infection and those that permit ongoing *Mtb* growth and dissemination.

Yet another tool for identifying and studying early infection is plasma metabolomics, in which small molecules are identified using high-resolution mass spectrometry. This approach has been successfully employed to identify direct metabolites of *Mtb*, and may be able to identify low levels even early in infection. Metabolomics has also been used to identify distinct signatures of the host immune response, whereby investigators were able to distinguish between risk-associated metabolites associated with a higher likelihood of progression to tuberculosis disease and disease-associated metabolites that increased in a time-dependent manner among those with progression to active tuberculosis disease [138–140]. Looking ahead, it will be important to determine whether these metabolic signatures are similarly predictive and valid in people with HIV.

## Conclusion

Across the scientific community, there is overwhelming consensus over the need to understand protective immunity to *Mtb* [55]. As a framework for considering early immunologic responses to *Mtb*, the mycobacterial set point provides a useful construct for understanding the impact of early immune control and clinical outcomes of tuberculosis. Given the disproportionate burden of tuberculosis disease and deaths among people with HIV despite widespread ART [23], it is imperative to study the unique impacts of HIV on *Mtb* infection and susceptibility. To date, research evaluating the effects of HIV on the alveolar macrophage response to *Mtb* has been relatively limited, particularly with regard to the critical early events that help to dictate the mycobacterial set point. While we believe that a higher mycobacterial set point is a key contributor to the increased morbidity among people with HIV, additional research is needed to better understand the mechanisms and biological pathways that may be involved in facilitating mycobacterial immune evasion. A greater understanding of the impact of HIV on these earliest responses to *Mtb* may lead to the development of novel interventions and host-directed therapies to reduce the persistent global burden of tuberculosis in this vulnerable population.

## Data Availability

Not applicable.
